# Ethyl Pyruvate Increases Post-Ischemic Levels of Mitochondrial Energy Metabolites: A ^13^C-Labeled Cerebral Microdialysis Study

**DOI:** 10.3390/metabo10070287

**Published:** 2020-07-13

**Authors:** Kevin H. Nygaard, Jesper F. Havelund, Troels H. Nielsen, Carl-Henrik Nordström, Nils. J. Færgeman, Frantz R. Poulsen, Jan Bert Gramsbergen, Axel Forsse

**Affiliations:** 1 Department of Neurosurgery, Odense University Hospital, University of Southern Denmark, Sdr. Boulevard 29, 5000 Odense C, Denmark; troels.nielsen@rsyd.dk (T.H.N.); carl-henrik.nordstrom@med.lu.se (C.-H.N.); frantz.r.poulsen@rsyd.dk (F.R.P.); Jon.Axel.Forsse@rsyd.dk (A.F.); 2 BRIDGE—Brain Researche—Inter-Disciplinary Guided Excellence, Institute of Clinical Research, University of Southern Denmark, Winsløwparken 19, 5000 Odense C, Denmark; 3 VILLUM Center for Bioanalytical Sciences, Department of Biochemistry and Molecular Biology, University of Southern Denmark, Campusvej 55, 5230 Odense M, Denmark; jhav@bmb.sdu.dk (J.F.H.); nils.f@bmb.sdu.dk (N.J.F.); 4 Institute of Molecular Medicine, University of Southern Denmark, 5000 Odense C, Denmark; jbgramsbergen@health.sdu.dk

**Keywords:** cerebral metabolism, labeled succinate, liquid chromatography—mass spectrometry, mitochondrial dysfunction, transient ischemia

## Abstract

Mitochondrial dysfunction after transient cerebral ischemia can be monitored by cerebral microdialysis (CMD) using changes in the lactate and pyruvate concentrations and ratio. Other metabolites associated with mitochondrial (dys)function are, e.g., tricyclic acid (TCA) and purine metabolites. Ethyl pyruvate (EP) is a putative neuroprotectant, supposedly targeting mitochondrial energy metabolism, but its effect on cerebral energy metabolism has never been described using microdialysis. In this study we monitored the metabolic effects of EP in the endothelin-1 (ET-1) rat model using perfusion with ^13^C-succinate and analysis of endogenous and ^13^C-labeled metabolites in the dialysates by liquid chromatography-mass spectrometry (LC-MS). Adult Sprague Dawley rats (*n* = 27 of which *n* = 11 were included in the study) were subjected to the microdialysis experiments. Microdialysis probes were perfused with ^13^C-labeled succinate (1 mM), and striatal dialysates were collected at 30 min intervals before induction of the insult, during intracerebral application of ET-1, and during intravenous treatment with either EP (40 mg/kg) or placebo, which was administered immediately after the insult. The rats were subjected to transient cerebral ischemia by unilateral microinjection of ET-1 in the piriform cortex, causing vasoconstriction of the medial cerebral artery. Monitoring was continued for 5 h after reperfusion, and levels of endogenous and ^13^C-labeled energy metabolites before and after ischemia-reperfusion were compared in EP-treated and control groups. Infarct volumes were assessed after 24 h. In both the EP-treated and placebo groups, ET-1-induced vasoconstriction resulted in a transient depression of interstitial glucose and elevation of lactate in the ipsilateral striatum. In the reperfusion phase, the concentrations of labeled malate, isocitrate, and lactate as well as endogenous xanthine were significantly higher in the EP-group than in the placebo-group: (mean ± SEM) labeled malate: 39.5% ± 14.9, *p* = 0.008; labeled isocitrate: 134.8% ± 67.9, *p* = 0.047; labeled lactate: 61% ± 22.0, *p* = 0.007; and endogenous xanthine: 93.9% ± 28.3, *p* = 0.0009. In the placebo group, significantly elevated levels of uridine were observed (mean ± SEM) 32.5% ± 12.7, *p* = 0.01. Infarct volumes were not significantly different between EP-treated and placebo groups, *p* = 0.4679. CMD labeled with ^13^C-succinate enabled detection of ischemic induction and EP treatment effects in the ET-1 rat model of transient focal cerebral ischemia. EP administered as a single intravenous bolus in the reperfusion-phase after transient cerebral ischemia increased de novo synthesis of several key intermediate energy metabolites (^13^C-malate, ^13^C-isocitrate, and endogenous xanthine). In summary, mitochondria process ^13^C-succinate more effectively after EP treatment.

## 1. Introduction

Cerebral microdialysis (CMD) is used in neuro-intensive care to monitor cerebral metabolism for the prevention of secondary injury in patients with traumatic brain injury and aneurysmal subarachnoid hemorrhage (aSAH) [1]. It has also been a valuable tool in cerebrovascular research for many years. 

Conventional enzymatic analysis can measure dynamic trends of glucose, lactate, pyruvate and the lactate-to-pyruvate ratio (LPR), enabling the detection of ischemic or hypoxic events. Ischemia-related metabolic disturbances, such as mitochondrial dysfunction can also be detected [2,3,4]. The method is quick and can be performed at the bedside, but it detects only a very limited number of metabolites. 

**Figure 1:**
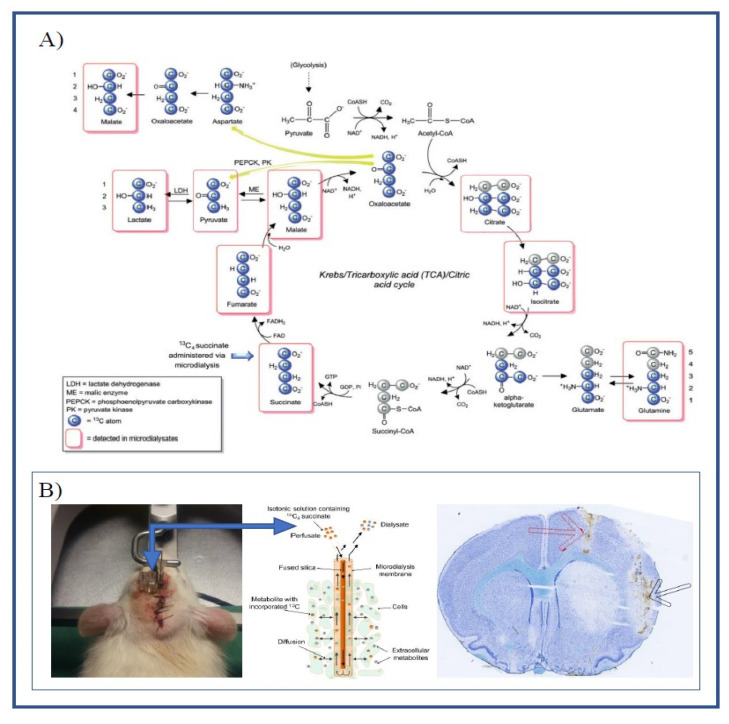
(**A**) Schematic illustration of the metabolism of ^13^C_4_ succinate via the TCA cycle and spin-out pathways. Blue-filled circles indicate ^13^C atoms. Red rectangular outlines indicate metabolites detected by LC-MS analysis. Modified from: Jalloh, Ibrahim et al. “Focally perfused succinate potentiates brain metabolism in head injury patients”. Journal of Cerebral Blood Flow and Metabolism 37, nr. 7 (July 2017): 2626–2638. (**B**) Illustrating the placement of guide cannulas in the left hemisphere (left) and brain histology using cryosectioning and Nissl staining (right), showing the placement of the microdialysis catheter in the striatum (black arrow) and catheter for ET-1 infusion (red arrow). Schematic of catheter illustrating the diffusion of labeled substrate and metabolites (middle) modified from: [10].

Metabolites measurements in dialysate samples using liquid chromatography coupled to mass spectrometry (LC-MS), a technique based on physical analyte separation and mass analysis, is much more demanding. However, it enables detection, identification and quantification of virtually any metabolite in the tricarboxylic acid (TCA) cycle, as well as many other compounds [5]. Thus studies have analyzed, not only intermediates in glycolysis and the TCA cycle, but also purines and purine derivatives associated with the pathophysiology of cerebral ischemia [6,7,8,9]. LC-MS in combination with microdialysis using a heavy carbon (^13^C) labeling marker may provide a detailed continuous measure of mitochondrial function in vivo (see Figure 1A). Furthermore, by comparing ratios of labeled and non-labeled metabolites, cellular leakage can be separated from de novo synthesis in intact mitochondria [10]. 

Neuroprotective treatment in cerebrovascular disease has been hampered by a low success rate in clinical trials [11]. This might be improved by focusing treatment on distinct metabolic states like post-ischemic mitochondrial dysfunction [4]. In the current study, we targeted the mitochondria-related metabolic disturbances that follow after transient cerebral ischemia with the putative neuroprotectant ethyl pyruvate (EP). EP is a simple derivative of pyruvate but is more stable in aqueous solutions and easily enters cells without the help of the monocarboxylate transporters, making it preferable in therapeutic applications [12,13]. Like pyruvate, EP is an effective scavenger of reactive oxygen species (ROS) and has been shown to exert anti-inflammatory properties [12,14]. Some studies suggest that EP might also serve as a substrate for the TCA cycle and may, thus, help to optimize ATP levels in the event of mitochondrial dysfunction [13,15,16].

The aim of this study was two-fold:

(a) To evaluate microdialysis of TCA metabolites (^12^C and ^13^C-labeled) and purine metabolites as tools to study mitochondrial dysfunction in the ET-1 model of cerebral ischemia-reperfusion injury.
(b) To assess whether EP-treatment ameliorates mitochondrial dysfunction (using microdialysis of TCA and purine metabolites) and/or reduces infarct size in the ET-1 rat model.

## 2. Results

### 2.1. Labeling Ratio 

The labeling ratio is a unique measurement associated with labeled microdialysis and is calculated as the ratio of labeled metabolites to endogenous metabolites. Table 1 shows the labeling ratios for various metabolites under baseline conditions. We observed large differences between metabolites, with the labeling ratio ranging from less than 0.01 and up to 11. Metabolites close to the labeling source in the TCA cycle (Figure 1A) had the highest labeling ratios.

### 2.2. Cerebral Microdialysis

**Figure 2:**
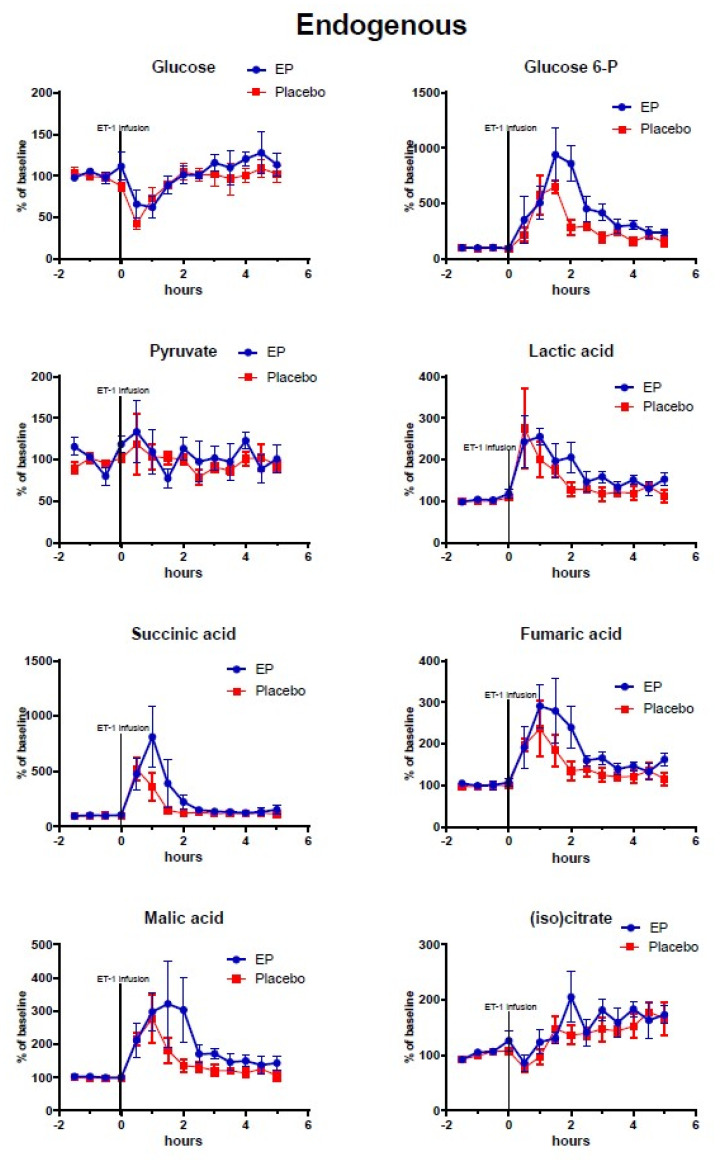
Changes of endogenous metabolites (non-labeled) in ipsilateral striatum after ethyl pyruvate (EP) and placebo treatment. Vasoconstriction of the medial cerebral artery (MCA) was induced by ET-1 infusion (150 pmol/15 µL/15 min) starting at time = 0 h. Blue symbols and line indicate data for rats treated with EP immediately after ET-1 infusion (*n* = 5). Red symbols and line indicate data for control rats (placebo treatment after ET-1, *n* = 6). Metabolite data are expressed as mean ± SEM of percentage change of each rat’s own baseline.

**Figure 3:**
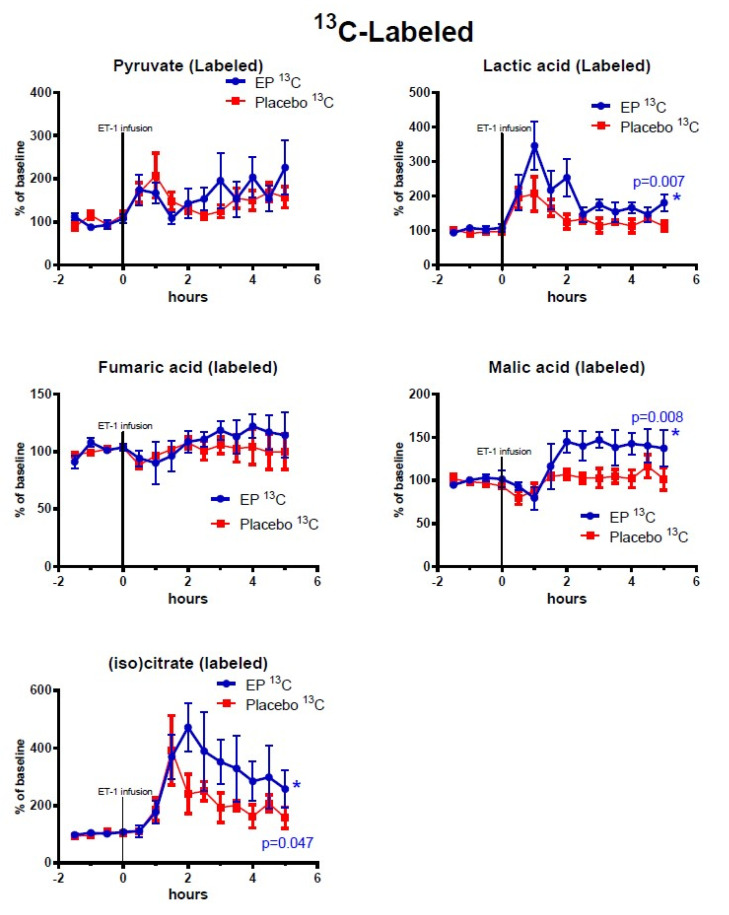
Changes of ^13^C-labeled metabolites in ipsilateral striatum after ethyl pyruvate (EP) and placebo treatment. Vasoconstriction of the medial cerebral artery (MCA) was induced by ET-1 infusion (150 pmol/15 µL/15 min) starting at time = 0 h. Blue symbols and line indicate data for rats treated with EP immediately after ET-1 infusion (*n* = 5). Red symbols and line indicate data for control rats (placebo treatment after ET-1, *n* = 6). Metabolite data are expressed as mean ± SEM of percentage change of each rat’s own baseline.

**Figure 4:**
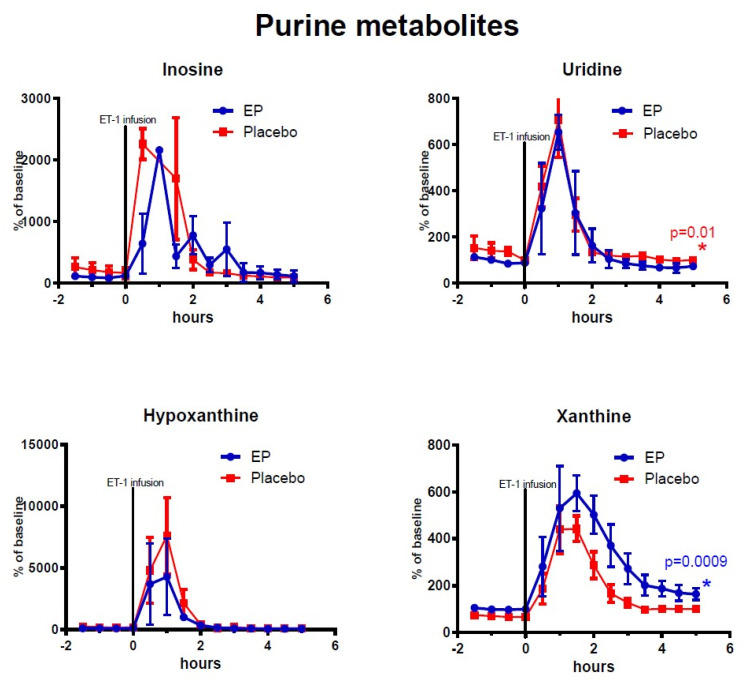
Changes of endogenous (non-labeled) purine metabolites in ipsilateral striatum after ethyl pyruvate (EP) and placebo treatment. Vasoconstriction of the medial cerebral artery (MCA) was induced by ET-1 infusion in the piriform cortex (150 pmol/15 µL/15 min) starting at time = 0 h. Blue symbols and line indicate data for rats treated with EP immediately after ET-1 infusion (*n* = 5). Red symbols and line indicate data for control rats (placebo treatment after ET-1, *n* = 6). Metabolite data are expressed as mean ± SEM of percentage change of each rat’s own baseline.

Figure 2, Figure 3, Figure 4 show the mean alterations in metabolite concentrations in the rats with ET-1 induced focal transient ischemia followed by either EP treatment or placebo. The endogenous (non-labeled) metabolites are shown in Figure 2, the ^13^C-labeled metabolites are shown in Figure 3, and the endogenous energy-related purine metabolites are shown in Figure 4. 

At time = 0 h micro-infusion of ET-1 was commenced and was accompanied by a drop in interstitial glucose concentration (EP: 52.04% ± 8.015, placebo: 47.98% ± 6.279, *p* = 0.7322) and rapid increases in endogenous glucose-6-phosphate and lactate levels. With a sampling frequency of 30 min, peak effects were seen between 0–2 h in endogenous lactate, succinate, fumarate, and malate, and a subsequent recovery phase began at 3 h with metabolite concentrations nearing baseline values. 

Among the TCA-cycle- and glycolysis-metabolites observed after reperfusion (time = 3–5 h) and treatment administration, the concentrations of labeled malate, lactate, and isocitrate were significantly higher in the EP-treatment group. Among the purine metabolites, only endogenous (non-labeled) xanthine displayed significantly higher concentrations in the EP-group compared to the placebo-group: (mean ± SEM) labeled malate: 39.5% points ± 14.9, *p* = 0.008; labeled isocitrate: 134.8% points ± 67.9, *p* = 0.047; labeled lactate: 61% points ± 22, *p* = 0.007; and endogenous xanthine: 93.9% points ± 28.3, *p* = 0.0009. However, Uridine had statistically significant higher concentrations in the placebo-group with an average of 32.5% points difference ±12.7, *p* = 0.01. Endogenous (non-labeled) glucose-6-phosphate, pyruvate, succinate, fumarate, malate, and citrate/isocitrate showed no significant difference, and neither did labeled pyruvate and fumarate. The purine metabolites inosine and hypoxanthine also showed no significant differences between the groups. 

### 2.3. Histology

Histological examination revealed striatal and cortical infarctions matching the intended ET-1 induced constriction of the proximal middle cerebral artery, confirming reliability of the experimental design. When comparing infarct volumes between EP-treatment and placebo groups, the variance was high and no significant differences in infarction volumes were observed: EP-treatment, mean infarction volume = 140.2 ± 51.25 (SEM), *n* = 5; and placebo, mean infarct volume = 95.73 ± 33.55 (SEM), *n* = 6, *p* = 0.4729.

## 3. Discussion

This study shows that ^13^C-labeled microdialysis with subsequent LC-MS analysis of dialysate fractions allows detailed monitoring of mitochondrial energy metabolism in the rat brain. Continuous perfusion with ^13^C-labeled succinate through the dialysis probe allowed detection and quantification of ^13^C-labeled TCA-cycle intermediates, indicating de novo synthesis, as well as endogenous (^12^C) TCA and purine metabolites, which result partly from ischemic cell death. Our approach also demonstrated increased TCA-cycle activity after the insult in rats treated with ethyl pyruvate.

### 3.1. The ET-1 Model of Focal Transient Cerebral Ischemia and Treatment Effects of Ethyl Pyruvate 

In the present study, 16 of 27 rats were excluded, because of insufficient hypoperfusion. The lack of effect of ET-1 is probably due to wrong placement of the injection cannula or occlusion of the injection cannula by a blood clot. The tip of the injection cannula must be placed in close vicinity to the proximal part of the medial cerebral artery (MCA), and the duration and severity of vasoconstriction of the MCA must be adequate to cause ischemic damage in the ipsilateral striatum. Hence, there are many factors involved resulting in various degree of brain damage in different rats. Unfortunately, because of insufficient vasoconstriction, a high number of rats were excluded, affecting the power of our study. However, we were able to remove the failed infarcts from both controls and EP-treated rats by using the microdialysis glucose data: Rats with less than 30% reduction in striatal glucose levels were defined as rats with insufficient hypoperfusion. Histological examination of the excluded rats showed no brain damage in ipsilateral striatum and thus confirmed the lack of effect of ET-1 in these rats. The ET-1-induced insults in the rats included in the study were of equal severity in the EP-treated and placebo-treated groups, as shown by similar changes in striatal lactate and glucose levels within the first hour after ET-1 infusion. 

Following transient cerebral ischemia, ^13^C-labeled and endogenous (^12^C) TCA intermediates were differentially affected in the EP-treated and control rats. Labeled TCA-cycle substrates can most likely only be produced in intact mitochondria, whereas interstitial levels of endogenous metabolites can be altered by disruption of organelles or cellular membrane integrity. This distinction is the basis for one of the main advantages of the labeled microdialysis technique [10].

The increased amounts of labeled malate, lactate, and isocitrate in the EP-group may be explained by stimulation of TCA-cycle and glycolysis activity by EP via various mechanisms, including EP providing acetyl-coA to enter the TCA cycle, or the conversion of pyruvate to lactate in the cytosol, which would transform NADH into NAD+ and help to maintain the large NAD+/NADH ratio needed for glycolysis [13,17]. Another possible explanation could be that EP serves as a ROS scavenger to optimize glycolysis, the TCA cycle, and purine metabolism as implied in previous studies [13,17,18,19]. 

### 3.2. Changes in Purine Derivatives

Purine derivatives are well-known markers of cerebral ischemic insult. Interstitial levels rise in response to deranged (predominantly astrocytic) neurotransmitter metabolism and ATP-breakdown [8,20]. Both detrimental and advantageous roles have been demonstrated for these intermediates, and several have been proposed as neuroprotective treatments [9,21,22]. Hypoxanthine is a purine derivative that is oxidized to xanthine by the enzyme xanthine oxidase (XO). Xanthine is thought to exert neuroprotective actions through further oxidation to uric acid, but the actions of XO itself contribute to formation of ROS [23]. In the current study, as expected, endogenous levels of all analyzed purine derivatives rose in response to ischemia. The response of hypoxanthine was especially pronounced as XO is inhibited by hypoxia. As reperfusion ensues, levels of purine metabolites begin to normalize, and XO is reactivated. Uridine, which is essential for neurocellular growth and repair, is upregulated under ischemic conditions, correlating with uric acid, and may help to alleviate ATP deficit [24,25,26]. The lower uridine values and higher xanthine values after reperfusion in the EP-treatment group may have contributed to attenuation of membrane degradation, and enhanced enzyme activity, respectively, possibly by the previously discussed ROS scavenging effects of EP [13].

### 3.3. Limitations of the Study—Variability of the ET-1 Model in Our Hands

In other experimental models of transient cerebral ischemia, such as the most common middle cerebral artery occlusion (MCAO) model, microdialysis experiments are carried out under general anesthesia [27]. Anesthesia affects brain energy metabolism and is thus a confounding factor when testing neuroprotective agents that are believed to improve energy metabolism [28]. By using the ET-1 model, we were able to induce cerebral ischemia, administer intravenous treatment, and perform continuous cerebral microdialysis in awake, freely moving animals. However, the combination of the ET-1 model and LC-MS analysis in the current study meant that the confirmation of hypoperfusion occurred after the experiment itself. The investigator thus had to rely on clinical signs of cerebral ischemia on the day of the experiment as laser Doppler or other methods of real-time CBF measurements were not available. The LC-MS analysis also requires larger microdialysis samples (30 µL), resulting in a lower time resolution (30 min intervals compared to 10–15 min with conventional enzymatic analysis). These methodological drawbacks resulted in a relatively large portion of animals not achieving the pre-specified criteria of transient ischemia. LC-MS analysis is sensitive, however, enabling differentiation of any chosen metabolite of a specific mass as well as detection of heavy-carbon labeling. Another drawback of LC-MS analysis is that pyruvate concentrations are difficult to measure. The pyruvate molecule is quite unstable in aqueous solution, and delayed concentration measurements show large variance. This makes calculating the lactate/pyruvate ratio difficult when using LC-MS analysis. 

The lack of any significant difference in infarction volumes may have been related to the timing of EP-administration or due to the rats being euthanized just 24 h after the insult. It could also be due to lack of power, because of the small sample size (*n* = 5 or 6). In the current study, the timing of administration was chosen for translational purposes (pre-reperfusion) and may be suboptimal regarding maximal treatment effect [16]. Previous studies have indicated that a different outcome in infarct size may be obtained with a longer recuperation period [29]. However, it may also be the case that the observed effects of EP on mitochondria-related metabolism in the acute phase is simply not favorable in terms of outcome, although the results of previous related studies suggest otherwise [13,14].

### 3.4. Clinical Implications of the Monitoring Method and Future Directions

Understanding the detailed temporal aspects of cerebral energy metabolism is important for clinical interventions. One example is the phenomenon of delayed neurological deterioration, a common complication in aSAH [2,30]. Earlier attributed solely to irritative vasospasm, it has now been shown to be associated with several other mechanisms amongst which mitochondrial dysfunction may represent a target for customized treatment [31]. In this pathologic metabolic state, which is often induced by prior ischemic events, mitochondrial function is insufficient, despite adequate amounts of substrate and oxygen, resulting in inefficient redox metabolism and ATP depletion, ROS production, and cell death [2,32]. 

A recent study with aSAH patients showed that a microdialytic pattern of mitochondrial dysfunction was actually more frequent than an ischemic pattern [2]. The ischemic pattern consists of an increased LPR due to increased amounts of lactate and decreased amount of pyruvate, whereas the pattern of mitochondrial dysfunction exhibits increased LPR with normal levels of pyruvate. 

As it is highly probable that mitochondrial dysfunction requires a different intervention strategy than traditional anti-vasospasm treatment, i.e., angioplasty and modified “triple H” (Hypertension, Hypervolemia and Hemodilution), its detection and differentiation from acute ischemia is highly relevant for the choice of clinical interventions in aSAH patients. 

Seeing that trace amount labeling of CMD presumably does not affect metabolism significantly and does not entail greater risk than conventional CMD. It may be considered in clinical observational studies where conventional CMD is used [32]. This would enable detailed investigations of mitochondrial dysfunction in relation to delayed neurological deterioration, as well as metabolic effects of presumed neuroprotective interventions in aSAH patients. Sample time collection could be increased or decreased to fit the needs of the researcher, seeing that only 15 µL are needed for the LC-MS analysis.

## 4. Materials and Methods 

The experiments were conducted from September 2017 to March 2018 at the animal facility of the Institute of Molecular Medicine, University of Southern Denmark, Odense. All experiments were approved by the local ethics committee and conducted in accordance with the Danish Animal Experiment Inspectorate and EU legislation (license no. 2017-15-0201-01256). 

The three-day experiment ran repeatedly, testing two rats at a time (randomized to treatment or placebo in drug trials). Adult Sprague Dawley rats (Taconic Biosciences A/S, Ejby, Denmark and Janvier labs, Saint-Berthevin, France) with mean weight 272.5 g (range 216–379 g) and mean age 7.5 weeks (range 6–9 weeks) were individually housed with a 12-h light/dark cycle and free access to food and water. 

All aspects related to the ET-1 rat model of transient focal cerebral ischemia, except labeled microdialysis and LC-MS, are described in detail in a previous publication from our group [16]. Briefly, the sedated animals underwent surgery and stereotaxic implantation of cerebral ET-1 and microdialysis probes and femoral vein catheter on Day 1 (Figure 1B). On Day 2, the awake rats were attached to a balanced swivel and connected to the dialysis setup. After 3 h of baseline sampling (30 min sampling frequency, flowrate 1.0 µL/min), ET-1 was infused unilaterally in close proximity to the middle cerebral artery at a rate of 1 µL/min for 15 min (150 pmol/15 µL/15 min). The rats were randomized to treatment or placebo administered immediately after induction of ischemia. The microdialysis samples were stored at −20 °C promptly after collection.

On Day 3 the rats were euthanized, and the brain was removed for histological investigation. 

### 4.1. Perfusion Fluid Labeling and LC-MS Analysis

^13^C-succinate labeling and LC-MS analysis were carried out as previously described, using sterile Ringer’s solution containing 1 mM ^13^C_4_ 99% succinate (Sigma-Aldrich Denmark A/S, Copenhagen, Denmark) as perfusion fluid [10]. LC-MS analysis was carried out as previously described in [10]. In brief, the lyophilized samples were resuspended in 12 µL 1% formic acid from which 2 µL were transferred to a pooled sample for quality control and annotation usage. A total of 10 µL were injected on the LC-MS system operated in negative ion mode. All reported annotations based on the existence of co-eluting fragments from the pooled sample analyzed in “all-ion” mode using 0, 10 and 40 V in collision energy (Metabolomics Standards Initiative level 3 annotation). Many more compounds were found than the reported. However, these could not be confidently annotated. Quality control samples were used to evaluate system reproducibility, and potentially, to exclude compounds with a relative standard deviation (RSD) above 30%, however, all shown compounds had a RSD < 15%. 

### 4.2. Randomization and Blinded Treatment Administration

The experiments were conducted using two rats at a time to ensure identical surgical preparation and procedures up to the point of randomization. Treatment was either ethyl pyruvate (40 mg/kg, 20 mg/mL ethyl pyruvate, Sigma-Aldrich Denmark A/S, Copenhagen, Denmark, diluted in sterile Ringer’s solution) or placebo (sterile Ringer’s solution) administered intravenously using the femoral vein catheter. Blinded randomization was conducted using masked vials with visually identical content and numbered 1 and 2. All animals were numbered with a tail marking and paired with a treatment number. Unblinding was performed at the completion of the experimental procedures. LC-MS analysis was evaluated with investigators blinded to treatment group assignment.

### 4.3. Statistical Analysis

Metabolite data are expressed as mean ± SEM of percentage change of each rat’s own baseline and are visualized as time-line graphs. With the time and resources available a total of 27 rats underwent the ET-1 experiment. After histological examination and LC-MS analysis, rats with insufficient hypoperfusion were excluded, thus, only rats with a transient decrease in glucose concentration of ≥30% in 30-min sampling were included. The full microdialysate LC-MS analysis was based on 11 rats: five EP rats and six placebo rats. Shapiro-Wilks’ test confirmed normal data distributions.

A mixed effects linear regression model for repeated measurements was used for statistical comparison of post-ischemic values, with time and group as random effect and individual specimen as fixed effect (STATA v 14.1, StataCorp., Texas, USA). 

### 4.4. Infarct Volumetry

**Figure 5:**
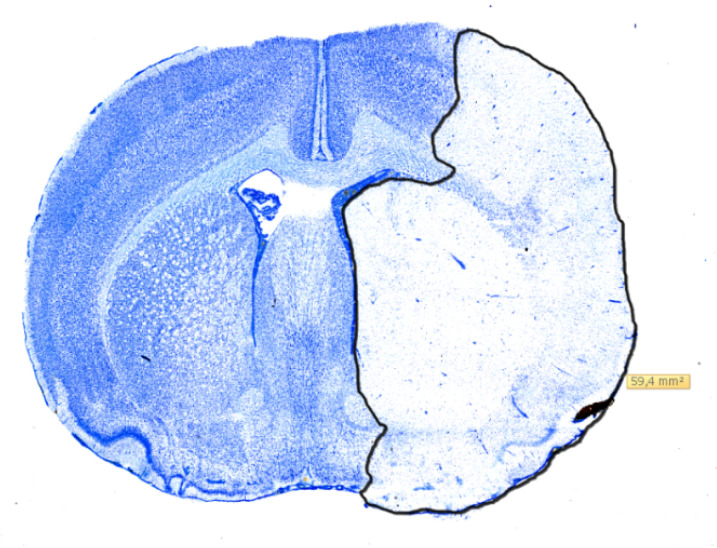
Digitalized coronal section of a rat brain stained with Toluidine-blue showing the measurement of infarcted area using NDP-view 2. The infarction has been outlined in black and the size is reported in mm^2^.

The whole rat forebrain was sectioned coronally into 30 µm slices, using a Leica CM 3050S cryostat, and placed on microscope slides. Next, they were stained in Toluidine-blue and digitalized using a pathoslide-scanner (Hamamatsu NanoZoomer 2.0-HT). This enabled digital measurement of the infarcted area using NDP-view 2 as seen in Figure 5 for each slide at a time. The cumulated infarct volume was then calculated using the cavalieri principle as mentioned in [16]. Histological comparison of infarction volumes was performed on the 11 randomized rats. As the Shapiro-Wilks’ test indicated non-normal data distributions, volumes were compared, using the non-parametric Mann-Whitney U test. *p*-values < 0.05 were considered significant.

## 5. Conclusions

Labeled microdialysis combined with LC-MS analysis offers a continuous method of in vivo monitoring for the detailed study of energy metabolism in transient cerebral ischemia. EP treatment affected purine metabolism and de novo production of selected mitochondrial metabolites, raising post-ischemic levels of TCA intermediates. All things considered, EP appeared to increase mitochondrial function, lower the production of ROS, and increase the rate of anabolic processes. In the current model, however, this did not lead to a reduction of infarct size compared to placebo. 

## Figures and Tables

**Table 1:** Ratios $\frac{labeled}{endogenous}$ of ^13^C-labeled metabolite versus endogenous (all ^12^C) metabolite under baseline conditions using perfusion with Ringer’s solution containing 1 mM ^13^C_4_-succinate (all 4 C-atoms ^13^C-labeled).

Metabolite	Labeling Ratio
Isocitrate (4 ^13^C, 2 ^12^C)	0.09
Lactate (3 ^13^C)	0.0029
Fumarate (4 ^13^C)	9.64
Pyruvate (3 ^13^C)	0.06
Malate (4 ^13^C)	11.43
